# Supplementary material for: The Impact of Research Results Feedback on the Lived Experiences of Elderly Participants in the DIMAMO Health Demographic Site: A Case of AWI-Gen Participants
Source: Int J Environ Res Public Health. 2025 Oct 15;22(10):1565. doi: 10.3390/ijerph22101565 (PMC12562761; doi:10.3390/ijerph22101565)
Supplement: Supplementary file 1 [file ijerph-22-01565-s001.zip › ijerph-3728506-supplementary.pdf]

Supplement Table S1

Table S1: Participants demographics.

Participant number	Gender	**Age**
1	Female	55-59
2	Female	60-64
3	Female	50-54
4	Female	60-64
5	Male	50-54
6	Female	55-59
7	Female	55-59
8	Male	70-74
9	Male	55-59
10	Male	65-69
11	Female	60-64
12	Male	70-74
13	Female	70-74
14	Female	60-64
15	Female	65-69
16	Female	60-64
17	Female	55-59
18	Male	65-69
19	Female	45-49
20	Female	55-59
21	Female	70-74
22	Female	60-64
23	Female	60-64
24	Female	75-79
25	Male	60-64
26	Male	50-54
27	Female	65-69

28	Male	55-59
29	Male	65-69
30	Female	45-49
31	Female	45-49
